# Comparing the associations between muscle strength, walking speed, and mortality in community-dwelling older adults of two birth cohorts born 28 years apart

**DOI:** 10.1007/s11357-023-00925-z

**Published:** 2023-09-01

**Authors:** Kaisa Koivunen, Erja Portegijs, Laura Karavirta, Taina Rantanen

**Affiliations:** 1 Faculty of Sport and Health Sciences and Gerontology Research Center, University of Jyväskylä, Jyväskylä, Finland; 2 Center of Human Movement Sciences, University of Groningen, University Medical Center Groningen, Groningen, the Netherlands

**Keywords:** Functional reserve capacity, Secular trends, Population-based, Physiological aging

## Abstract

Reduced age-specific mortality and increased muscle strength and walking speed of current older adults may have altered the relationships between these factors as more people may be above the reserve capacity threshold. We compared the cross-sectional associations between muscle strength and walking speed, and the associations of muscle strength and walking speed with five-year mortality between two population-based cohorts of 75- and 80-year-old people born 28 years apart. Maximal isometric grip and knee extension strength and walking speed were measured in 2017–2018 (*n* = 726). Mortality was ascertained from registers. The associations were compared with data of same-aged people studied in 1989–1990 with identical protocols (*n* = 500). The knee extension strength-walking speed relationship showed plateauing at higher strength levels among the later-born men, whereas the earlier-born men and women of both cohorts with lower strength levels were on the linear part of the curve. In the later-born women with lower five-year mortality rate (1.16 vs. 5.88 per 100 person-years), the association between grip strength and mortality was markedly different from the earlier cohort (HR 1.13 [95% CI 0.47–2.70] vs. 0.57 [0.37–0.86]). For knee extension strength and walking speed, the mortality hazards were similar between the cohorts, although statistically non-significant in the later-born women. In men, the later-born cohort showed similar associations as observed in the earlier-born cohort despite having lower mortality rate (2.93 vs. 6.44). Current older adults have more functional reserve that will likely help them to maintain walking ability for longer while also contributing to better survival.

**Supplementary Information:**

The online version contains supplementary material available at 10.1007/s11357-023-00925-z.

## Introduction

Muscle strength and walking speed decline in old age and reflect life-long exposures to beneficial and detrimental external influences, genetic predispositions, diseases and aging itself, making them powerful predictors of future health changes [1]. A certain minimum level of muscle strength is required for walking, above which muscle strength and walking speed correlate positively up to a level where strength is not a limiting factor anymore. Beyond this level, a reserve of strength is present and protects from walking decline [2–5]. Walking speed integrates information of functioning of multiple physiological subsystems, including musculoskeletal but also e.g., cardiovascular and nervous systems underlying ability to move [6]. Thus, good muscle strength and walking speed both reflect intrinsic capacity that protects individuals from progressive health decline during aging, and they may be particularly beneficial for surviving and recovering after adverse health events, such as bone fractures [7].

We and other researchers have recently reported that with increased life expectancy, more recently born cohorts of older adults have greater muscle strength and walking speed than the same-aged individuals had earlier. For example, Strand et al. [8] and Tomkinson et al. [9] reported improved hand grip strength in Norwegian and Japanese older adults. We found in Finland that a more recent birth cohort had better muscle strength and walking speed at ages 75 and 80 compared to the same-aged cohort 28 years earlier [10]. These positive trends are most likely a result of overall improvements in living conditions and more favorable life-course exposures at earlier stages of life. However, it remains unclear, what impact these birth cohort differences in functional capacity may have on the associations between muscle strength, walking ability and mortality.

Current evidence concerning the role of muscle strength and walking speed as predictors of future health outcomes in older adults is mainly based on prospective cohort studies with data collected in the past when average life span was meaningfully shorter, and participants at the age they were studied had a shorter life expectancy than people of the same age do today. To the best of our knowledge, no previous studies have investigated whether the improved muscle strength, walking speed, and reduced mortality risk of later born older adults might alter their relationships. It is possible that the associations have been attenuated as a higher proportion of the later born cohorts are above the expected reserve capacity levels, and consequently their risks for mobility limitations and health decline are lower than was in the same age in the past. However, it is also possible that higher muscle strength is needed for voluntary body movements. Older adults nowadays are taller and heavier than before, most likely due to better nutrition during earlier life phases [10] and consequently more strength will be needed to carry and move the higher body mass [11]. It is also possible that socio-economic development may underlie the associations of muscle strength with health decline. In a comparative study by Oksuzyan et al. [12] the strongest prognostic value of grip strength for mortality was seen among the population group with worst health and most disadvantaged economic situation. Having physiological reserve indicated by higher muscle strength may become less crucial when living conditions are more favorable. The comparison of the associations and prognostic importance physical performance measurements across socio-historically or socio-economically distinct settings is, however, difficult due to challenges in ensuring the comparability of the assessment methods and populations studied.

Two population-based studies conducted at our research center 28 years apart using the same standardized assessment methods and comprehensive Finnish mortality registry data provide us with a robust setting to explore the potential secular changes in the associations between muscle strength, walking speed and mortality. We compared the associations of maximum grip and knee extension strength with current walking speed and the associations of grip strength, knee extension strength and walking speed with five-year mortality between these two birth cohorts of older adults both aged 75 and 80 at the time of the assessments.

## Methods

### Study participants

The present analyses draw on data from two cohort studies conducted at the University of Jyväskylä, Finland. Of the earlier born Evergreen cohort, the participants were born in 1910 and 1914 and examined in 1989–1990 [13]. In the later born Evergreen II cohort, the participants were born in 1938–1939 and 1942–1943 and examined in 2017–2018 as part of the *Active Ageing – Resilience and external support as modifiers of disablement outcome* (AGNES) project [14]. Both samples were recruited from the Finnish Population Register based on birth year and place of residence and target group consisted of all community-dwelling 75- and 80-year-old residents of the city of Jyväskylä. The recruitment processes in both cohorts followed similar procedures and were as inclusive as possible.

All participants in both cohorts signed an informed consent, and the research ethics principles required at the time were followed. The ethical committee of the Central Finland Hospital gave a positive statement of the Evergreen II study on August 23, 2017. In both data collections, the participants were interviewed at their homes, after which the functional assessments were scheduled in the research center of the University of Jyväskylä. In Evergreen, 500 (77%) and in Evergreen II, 726 (40%) of those eligible participated in both the home interviews and laboratory assessments.

Our previous analyses showed that there were no significant differences between cohorts in non-participants’ self-rated health or reported reasons for not participating in the study suggesting that the cohorts were comparable [10]. A more detailed overview of the design and information on participation and non-participation is has been reported elsewhere [10].

### Muscle strength measurements

The methods and procedures of maximal isometric grip strength and knee extension strength were identical for the different cohorts. In the Evergreen II cohort both strength measurements were performed using an adjustable dynamometer chair (Good Strength; Metitur Oy, Palokka, Finland) and the result expressed in Newtons (N) [15]. In the earlier Evergreen cohort, a prototype of the Good Strength device incorporating equal strain gauge technology was used with identical joint angle settings and instructions to the participant. The measurements were performed on the side of the dominant hand in a sitting position with the lower back supported. Hand grip strength was measured using a dynamometer fixed to the arm of the chair. Knee extension strength was measured at an angle of 60 degrees from the fully extended leg towards flexion. After a practice trial, the test was performed at a minimum of three times with a 1-min rest period between the trials until no further improvement occurred, and the highest value in Newtons was recorded [14]. Both strength tests have shown excellent test–retest reliability. Among 80-year-old participants, the Pearson correlation coefficients between measurements conducted one to two weeks apart were r = 0.967 for hand grip strength and r = 0.965 for knee extension strength [15].

### Outcomes

*10-m maximal walking speed* was measured in the laboratory corridor with a hand-held stopwatch. Five meters were allowed for acceleration, and the participant was instructed to continue for a few meters after crossing the finish line [14].

Death dates were obtained from the population register of Finland. The participants were followed up for mortality from the date of the home interview until the first of August 1994 in the Evergreen cohort and until the first of August 2022 in the Evergreen II cohort.

### Covariates and background variables

We chose correlates, which differed between the two birth cohorts, and in theory, may be part of the mechanism underlying potential birth cohort differences in the associations between muscle strength, walking speed and mortality. Body size, especially height, has been shown to affect both muscle strength and walking speed [16] and was measured with a stadiometer in centimeters. Weight was measured with a beam scale in kilograms. Body mass index (BMI) was calculated by dividing the body mass by square of the body height and is expressed in units of kg/m^2^. Cognitive functioning was assessed with the Digit Symbol coding task, which measures processing speed and short-term visual memory (Wechsler Adult Intelligence Scale-Revised) [17]. The participant drew the correct symbols below their equivalent numbers by using a number-to-symbol coding key. The test time limit was 90 s, and the score is the number of correct symbols in the correct order (maximum 65). Socio-economic status was indicated by full-time education in years. Lifestyle factors were described with physical activity and daily smoking. Physical activity was assessed with a self-reported validated item with six response options ranging from “mostly sitting and resting” to “regular strenuous exercise” [15] and for the analyses, the responses were recoded as low, moderate and high physical activity. Smoking was coded as never vs. currently/earlier in life.

### Statistical analyses

Descriptive statistics were summarized as means with standard deviations (SD) or frequencies with percentages, as appropriate. Two-tailed t-test and chi-square test were used for group comparisons for continuous and categorical variables, respectively. The relationship between muscle strength and walking speed has been suggested to be curvilinear, whereby muscle strength has a stronger impact on walking speed at lower levels of muscle strength [3, 4]. Therefore, multiple regression analysis was used to test both linear and curvilinear relationships between muscle strength and walking speed, and cohort differences in these associations. First, we entered the main effect (linear term) of the muscle strength in the first model and then in the second model, the curvilinear effect (quadratic term) for muscle strength first separately for the birth cohorts and then both cohorts combined including the cohort variable as a covariate. We report unadjusted and adjusted models including body height and weight, education, physical activity, smoking, and coding task as covariates. We also assessed whether the associations differed between the cohorts by testing birth cohort-by-muscle strength interactions when the birth cohorts were combined. Fit of the models was assessed by examining the statistical significance of the linear, quadratic and interaction terms’ coefficient and comparing the adjusted R-squared between the models.

Cox regression analysis was used to examine the associations of muscle strength and walking speed with 5-year mortality in separate models. First, we examined the cohorts separately and then both cohorts combined including the cohort variable as a covariate. In the analyses combining both cohorts, birth cohort-by-muscle strength and birth cohort-by-walking speed interactions were studied to test whether the differences in the strength of the associations were statistically significant between the cohorts. The first model was adjusted for age group. Subsequently, we run several models adding covariates one or two at a time to the age adjusted model. The model 2 was adjusted for age, weight and height, the model 3 for age and education, model 4 for age and coding task and finally, model 5 for age, physical activity, and smoking. The proportional hazards assumption was checked using the Schoenfeld residuals.

All the analyses were conducted separately for men and women. No data were excluded from the analyses. Data normality was assessed with q-q plots. Statistical significance for all the statistical models was accepted at *p* < 0.05. Group comparisons were analyzed using SPSS statistical software (Chicago, IL) version 26 and regression models in R (version 4.2.1) The qgraph (v. 1.9.5) package was used for visualization of the regression curves and the survival package (v. 3.5–5) for Cox regression models.

## Results

In the earlier born cohort, 36 men and 67 women died during 559 and 1 140 person-years of surveillance, respectively. In the later born cohort, 37 men and 20 women died in their respective 1261 and 1715 years of follow-up. The crude mortality rates were lower in the later than earlier born cohorts: in men 2.93 per 100 person-years vs. 6.44, and in women 1.16 vs. 5.88, respectively. The average ages at death did not differ between the cohorts (Table 1).

**Table 1:** Characteristics of the two birth cohorts assessed in 1989–1990 vs. 2017–2018 and stratified by the vital status after the 5-year follow-up

	Men (*n* = 556)				Women (*n* = 854)			
	Alive	Deceased	B/w vital status	B/w deceased in two cohorts	Alive	Deceased	B/w vital status	B/w deceased in two cohorts
Grip strength, N	Mean (SD)	Mean (SD)	*p*-value	*p*-value	Mean (SD)	Mean (SD)	*p*-value	*p*-value
1989–1990	363.0 (84.5)	304.5 (102.8)	**0.001**	**0.039**	209.0 (60.7)	182.7 (68.9)	**0.003**	** < 0.001**
2017–2018	393.1 (75.8)	350.5 (75.7)	**0.002**		229.0 (51.0)	233.0 (42.4)	0.731	
KE strength, N								
1989–1990	361.6 (82.9)	311.6 (112.3)	**0.006**	0.157	226.9 (70.2)	186.6 (80.2)	** < 0.001**	** < 0.001**
2017–2018	438.3 (99.0)	352.9 (121.0)	** < 0.001**		293.8 (82.0)	257.0 (79.3)	0.050	
Walking speed, m/s								
1989–1990	1.7 (0.5)	1.4 (0.6)	**0.002**	0.264	1.4 (0.4)	1.2 (0.4)	** < 0.001**	** < 0.001**
2017–2018	1.9 (0.4)	1.6 (0.5)	** < 0.001**		1.7 (0.3)	1.6 (0.4)	0.079	
Height, cm								
1989–1990	169.5 (6.1)	169.0 (6.9)	0.723	0.086	155.8 (5.4)	155.2 (5.8)	0.407	**0.002**
2017–2018	172.7 (6.0)	171.7 (6.0)	0.332		158.9 (5.2)	158.9 (6.1)	0.382	
Weight, kg								
1989–1990	75.1 (10.8)	72.6 (13.6)	0.247	**0.009**	66.4 (11.0)	65.7 (11.4)	0.671	**0.016**
2017–2018	80.1 (12.6)	81.5 (14.8)	0.536		70.4 (12.0)	73.7 (16.3)	0.240	
BMI, kg/m^2^								
1989–1990	26.1 (3.5)	25.4 (4.4)	0.269	**0.021**	27.3 (4.4)	27.3 (4.6)	0.939	0.215
2017–2018	26.9 (4.0)	27.7 (5.1)	0.349		27.9 (4.7)	29.0 (7.3)	0.321	
Coding task, 0–65								
1989–1990	22.4 (9.0)	15.5 (10.1)	** < 0.001**	** < 0.001**	21.4 (9.8)	15.7 (7.7)	** < 0.001**	** < 0.001**
2017–2018	31.4 (9.8)	26.1 (10.7)	**0.003**		34.1 (9.6)	32.9 (11.8)	0.587	
Education, years								
1989–1990	6.1 (3.6)	6.3 (4.2)	0.725	** < 0.001**	6.0 (3.3)	5.9 (2.7)	0.835	** < 0.001**
2017–2018	12.3 (4.3)	10.7 (4.6)	**0.049**		11.9 (5.0)	12.8 (4.5)	0.434	
Age group, 75 vs. 80 years	**n (%)**	**n (%)**			**n (%)**	**n (%)**		
1989–1990	85 (66)	19 (53)	0.134	0.733	160 (60)	31 (46)	0.051	0.141
2017–2018	162 (58)	21 (57)	0.861		238 (61)	13 (65)	0.712	
Age at death								
1989–1990	**–**	79.8 (2.6)	**–**	0.927	**–**	80.1 (2.5)	–	0.798
2017–2018	**–**	79.3 (2.1)	**–**		**–**	79.9 (2.7)	–	
Physical activity								
1989–1990								
Low	37 (29)	17 (47)	0.109	**0.021**	63 (24)	27 (42)	**0.010**	0.198
Moderate	78 (61)	17 (47)			196 (74)	35 (55)		
High	13 (10)	2 (6)			7 (3)	2 (3)		
2017–2018								
Low	19 (7)	6 (17)	0.085		46 (12)	4 (20)	0.397	
Moderate	197 (71)	24 (69)			295 (76)	15 (75)		
High	60 (22)	5 (14)			47 (12)	1 (5)		
Smoking status, yes								
1989–1990	80 (66)	22 (67)	0.953	0.395	22 (8)	6 (9)	0.823	0.191
2017–2018	130 (48)	21 (57)	0.297		67 (17)	4 (20)	0.753	

*B/w* between, *KE* knee extension, *N* Newton, *cm* centimeters, *m/s* meters per second. In the 1989–1990 cohort, men *n* = 164 and women *n* = 392; in the 2017–2018 cohort, men *n* = 392 and women *n* = 518

Statistically significant values (*p* <.05) are shown in bold

The baseline characteristics of the participants according to their vital status after the five-year follow-up are shown in the Table 1. In general, those who died during the 5-year follow-up, had worse functional status at baseline assessment in terms of muscle strength, walking speed and cognitive coding task when compared to survivors in both cohorts. However, among the later born women, the group differences were less clear and statistically nonsignificant. In addition, among the later born men, the deceased had shorter full-time education. Among the earlier born women, the deceased were less physically active compared to survivors.

Furthermore, we analyzed whether the deceased in two birth cohorts differed from each other in their characteristics. The deceased of the later born cohort had better functional status in terms of muscle strength, walking speed and coding test, and were taller and heavier than the deceased of the earlier born cohort. The deceased of the later born cohort also had longer education and were more physically active (only men) than the deceased in the earlier cohort.

### Associations between muscle strength and walking speed

**Fig. 1:**
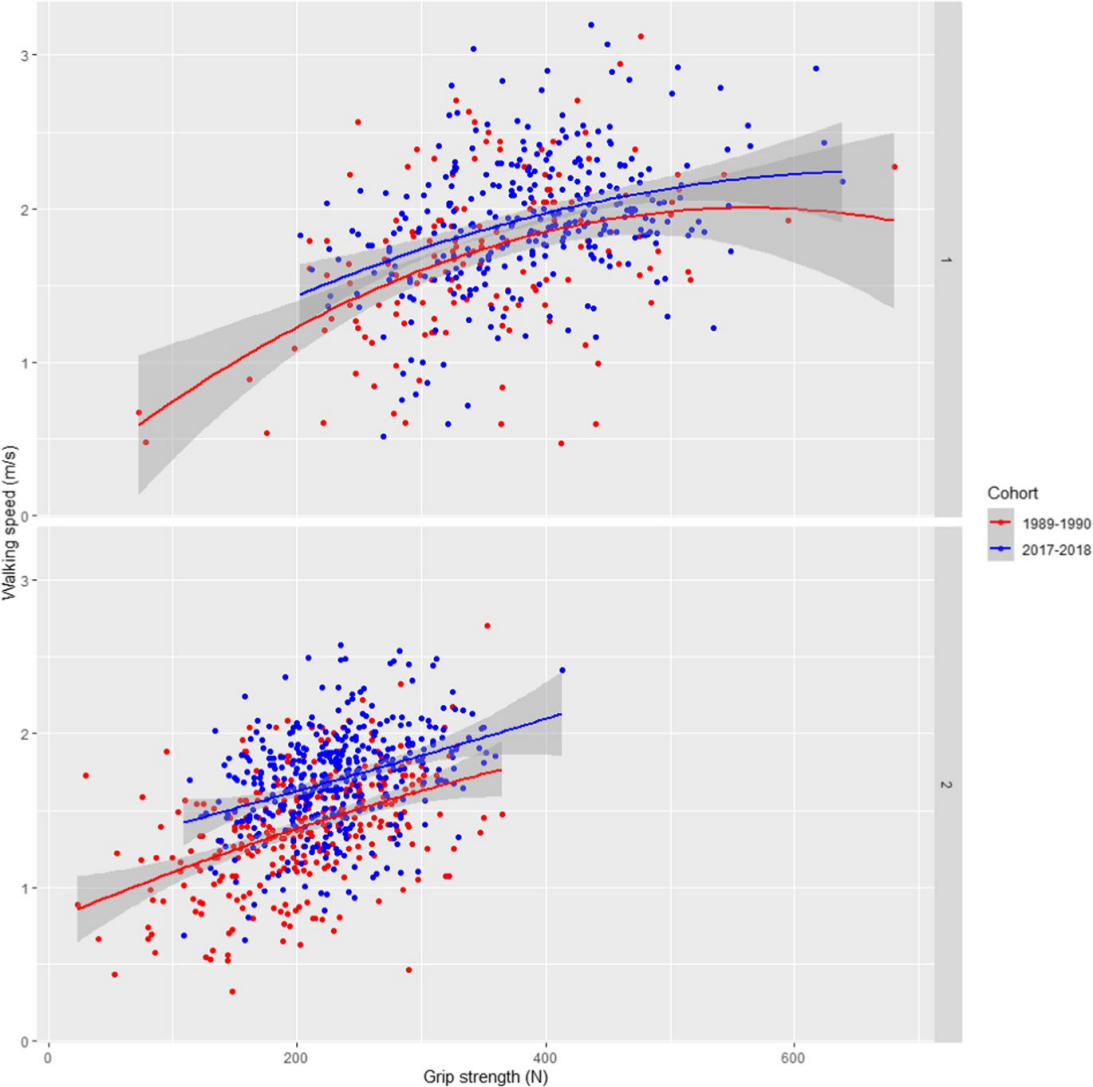
Unadjusted quadratic regression curves of the association between grip strength and walking speed in men and women of the two birth cohorts. The gray band shows the 95% confidence interval for the regression line. In the 1989–1990 cohort, men *n* = 164 and women *n* = 392; in the 2017–2018 cohort, men *n* = 392 and women *n* = 518

**Fig. 2:**
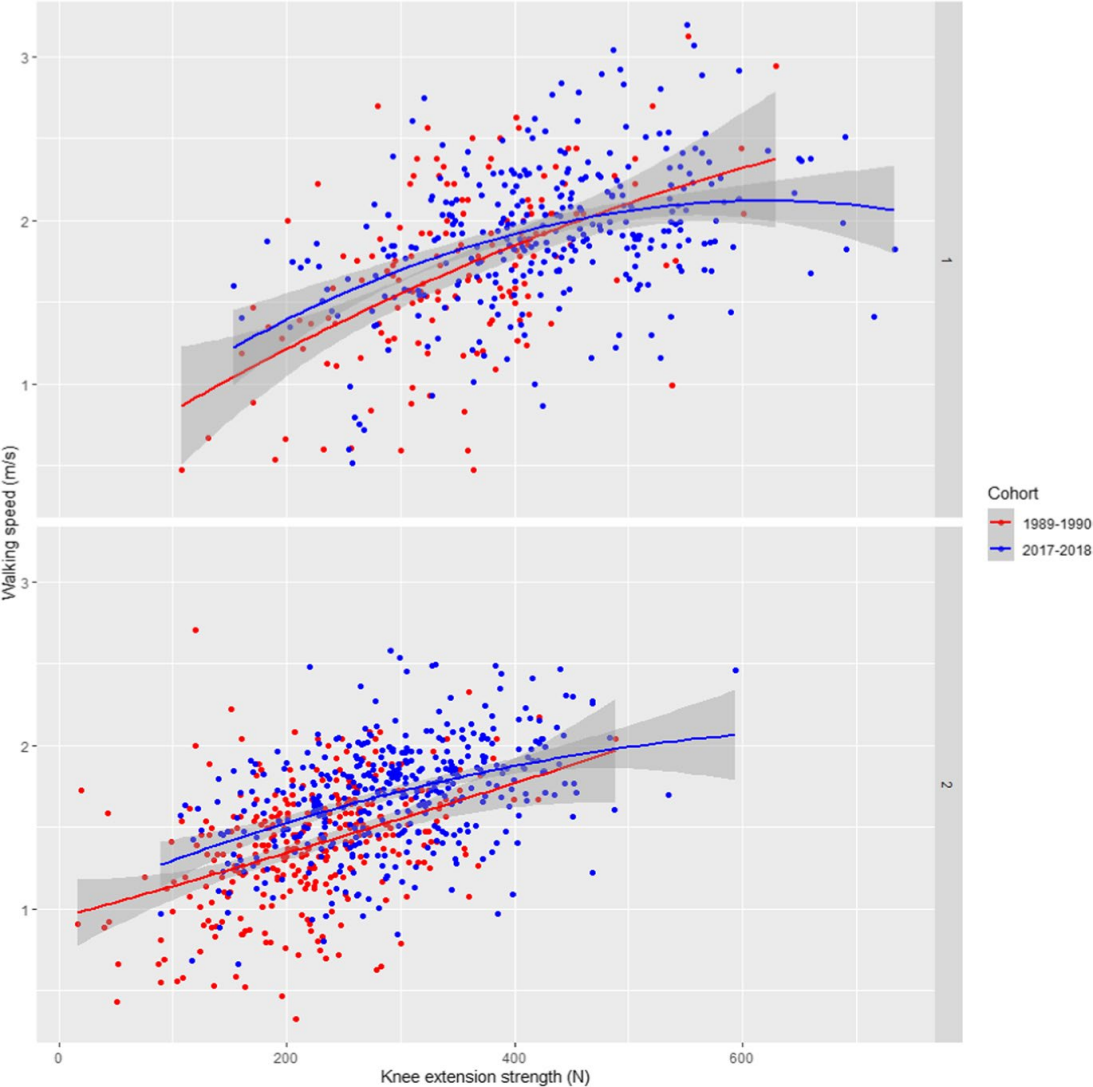
Unadjusted quadratic regression curves of the association between knee extension strength and walking speed in men and women of the two birth cohorts. The gray band shows the 95% confidence interval for the regression line. In the 1989–1990 cohort, men *n* = 164 and women *n* = 392; in the 2017–2018 cohort, men *n* = 392 and women *n* = 518

Overall, better grip and knee extension strength were associated with faster walking speed in both sexes and birth cohorts (Tables 2 and 3). Especially in women, there was a clear group level difference between the birth cohorts in the associations between muscle strength and walking speed mostly without overlapping confidence intervals indicating that the same amount of muscle strength translated into faster walking speed in the later born cohort compared to those born earlier (Figs. 1 and 2). The constant of the regression line, i.e., the estimated value of walking speed where the line crosses the y-axis, was greater among the later than the earlier born cohort in the unadjusted models (grip strength: 1.164 vs. 0.844 and knee extension strength: 1.185 vs. 0.927). However, the differences attenuated or vanished after adjustment for covariates.

**Table 2:** Linear and curvilinear associations between grip strength and walking speed stratified by birth cohort

		2017–2018 cohort				1989–1990 cohort			
		Unadjusted		Adjusted		Unadjusted		Adjusted	
Men		B (SE)	*p*	B (SE)	*p*	B (SE)	*p*	B (SE)	*p*
M1	Constant	1.141 (0.117)		1.267 (0.170)		0.845 (0.153)		1.213 (0.250)	
	Linear term	0.202 (0.029)	** < 0.001**	0.170 (0.029)	** < 0.001**	0.241 (0.042)	** < 0.001**	0.116 (0.045)	**0.012**
	***R***^***2***^	*0.130*		*0.350*		*0.171*		*0.405*	
M2	Constant	0.605 (0.423)		0.626 (0.719)		0.138 (0.353)		0.774 (0.981)	0.432
	Linear term	0.485 (0.217)	**0.026**	0.368 (0.200)	0.067	0.663 (0.195)	**0.001**	0.376 (0.180)	**0.039**
	Quadratic term	-0.035 (0.027)	0.188	-0.025 (0.025)	0.319	-0.059 (0.027)	**0.028**	-0.035 (0.024)	0.138
	***R***^***2***^	*0.132*		*0.350*		*0.192*		*0.412*	
Women									
M1	Constant	1.164 (0.072)		1.404 (0.415)		0.844 (0.063)		1.434 (0.481)	
	Linear term	0.230 (0.031)	** < 0.001**	0.215 (0.028)	** < 0.001**	0.265 (0.030)	** < 0.001**	0.192 (0.031)	** < 0.001**
	***R***^***2***^	*0.121*		*0.405*		*0.199*		*0.421*	
M2	Constant	1.188 (0.253)		1.304 (0.471)		0.777 (0.139)		1.367 (0.490)	
	Linear term	0.209 (0.216)	0.333	0.296 (0.182)	0.104	0.339 (0.140)	**0.016**	0.280 (0.021)	**0.027**
	Quadratic term	0.004 (0.045)	0.922	-0.017 (0.038)	0.652	-0.019 (0.034)	0.588	-0.021 (0.030)	0.470
	***R***^***2***^	*0.118*		*0.403*		*0.197*		*0.420*	

M1 (model 1) includes linear term of grip strength, M2 (model 2) includes linear + quadratic terms of grip strength, B = unstandardized regression coefficient (per -100 Newtons in grip strength), SE = standard error, R^2^ adjusted, the variance explained by the model, covariates in the adjusted models: age group, height, weight, education, physical activity, smoking, and coding task. In the 1989–1990 cohort, men *n* = 164 and women *n* = 392; in the 2017–2018 cohort, men *n* = 392 and women *n* = 518

Statistically significant values (*p* <.05) are shown in bold

**Table 3:** Linear and curvilinear associations between knee extension strength and walking speed stratified by birth cohort

		2017–2018 cohort				1989–1990 cohort			
		Unadjusted		Adjusted		Unadjusted		Adjusted	
Men		B (SE)	p	B (SE)	p	B (SE)	p	B (SE)	*p*
M1	Constant	1.260 (0.096)		0.536 (0.601)		0.664 (0.145)		0.735 (0.930)	
	Linear term	0.155 (0.022)	** < 0.001**	0.123 (0.021)	** < 0.001**	0.293 (0.040)	** < 0.001**	. 176 (0.041)	** < 0.001**
	**R**^**2**^	0.140		0.351		0.260		0.450	
M2	Constant	0.538 (0.273)		-0.309 (0.644)		0.416 (0.360)		0.767 (0.942)	
	Linear term	0.510 (0.128)	** < 0.001**	0.511 (0.119)	** < 0.001**	0.440 (0.200)	**0.029**	0.129 (0.199)	0.520
	Quadratic term	-0.041 (0.015)	**0.005**	-0.044 (0.013)	**0.001**	-0.020 (0.027)	0.453	0.006 (0.026)	0.807
	***R***^***2***^	*0.159*		*0.374*		*0.258*		*0.446*	
Women									
M1	Constant	1.185 (0.055)		1.119 (0.401)		0.927 (0.060)		1.338 (0.475)	
	Linear term	0.174 (0.018)	** < 0.001**	0.154 (0.017)	** < 0.001**	0.208 (0.026)	** < 0.001**	0.174 (0.025)	** < 0.001**
	***R***^***2***^	*0.184*		*0.441*		*0.169*		*0.433*	
M2	Constant	1.027 (0.143)		0.974 (1.442)		0.943 (0.118)		1.357 (0.005)	
	Linear term	0.289 (0.097)	**0.003**	0.256 (0.506)	0.613	0.192 (0.145)	0.067	0.135 (0.089)	0.130
	Quadratic term	-0.019 (0.016)	0.230	-0.017 (0.014)	0.217	0.003 (0.023)	0.875	0.008 (0.019)	0.652
	***R***^***2***^	*0.185*		*0.441*		*0.166*		*0.431*	

M1 (model 1) includes linear term of knee extension strength, M2 (model 2) includes linear + quadratic terms of knee extension strength, B = unstandardized regression coefficient (per -100 Newtons in knee extension strength), SE = standard error, R^2^ adjusted, the variance explained by the model, covariates in the adjusted models: age group, height, weight, education, physical activity, smoking, and coding task. In the 1989–1990 cohort, men *n* = 164 and women *n* = 392; in the 2017–2018 cohort, men *n* = 392 and women *n* = 518

Statistically significant values (*p* <.05) are shown in bold

We analyzed whether the association between muscle strength and walking speed was curvilinear by adding a quadratic term in the regression models. For maximal hand grip strength, only for the earlier born cohort of men the quadratic term was significant and adding it in the regression models increased the coefficient of determination. This suggests that the association was curved and plateaued at the higher strength levels. For women, or for later born men, adding a quadratic term in the models did not materially change them. Adding other covariates in the model attenuated the slope of the grip strength with walking speed suggesting that within cohorts, differences in age group, height, weight, education, physical activity, smoking, and coding task result explained some but not all the observed statistically significant associations. The strength of the grip strength-walking speed association did not differ between the cohorts for either sex (Supplemental Table 1).

For knee extension strength, only for the later born cohort of men quadratic term was significant demonstrating a plateauing association with walking speed at higher strength levels (Table 3, Fig. 2). Similar was not observed in the earlier born cohort of men or in women of either cohort. Adding covariates in the models did not materially change the association of knee extension strength and walking speed in the later born cohorts of men or women but did so in the earlier born cohorts especially in men. However, in men, the unadjusted knee extension strength-by-cohort interaction was statistically significant (B -0.243, SE 0.074, *p* = 0.001; Supplemental Table 2), most likely due to the flattening curve in the later born men. The interaction remained statistically significant after adjustment for covariates.

### The associations between muscle strength and 5-year mortality

The associations of grip and knee extension strength and walking speed with mortality are presented in Table 4 for men and Table 5 for women. In the age adjusted models, the mortality hazard reduction per 100N increase in grip and knee extension strength and 0.1 m/s in walking speed was slightly greater among the later than the earlier born men. The associations also remained relatively unchanged after adjustments for covariates but in the earlier born men, the associations of knee extension strength and walking speed with mortality vanished after adjustment for the coding task.

**Table 4:** Associations of muscle strength and walking speed with 5-year mortality in two birth cohorts among men

	2017–2018 cohort	1989–1990 cohort
	HR (95% CI)	HR (95% CI)
Grip strength, per 100 N		
M 1: Adjusted for age	**0.45 (0.28–0.73)**	**0.52 (0.34–0.78)**
M 2: Adjusted for age, height, and weight	**0.44 (0.26–0.72)**	**0.45 (0.27–0.76)**
M 3: Adjusted for age and education	**0.48 (0.29–0.79)**	**0.53 (0.35–0.79)**
M 4: Adjusted for age and coding task	**0.50 (0.31–0.82)**	**0.65 (0.44–0.98)**
M 5: Adjusted for age, PA, and smoking	**0.52 (0.31–0.86)**	**0.55 (0.35–0.87)**
Knee extension strength, per 100 N		
M 1: Adjusted for age	**0.43 (0.31–0.61)**	**0.55 (0.37–0.84)**
M 2: Adjusted for age, height, and weight	**0.42 (0.30–0.61)**	**0.55 (0.35–0.88)**
M 3: Adjusted for age and education	**0.46 (0.32–0.65)**	**0.58 (0.38–0.89)**
M 4: Adjusted for age and coding task	**0.49 (0.34–0.69)**	0.70 (0.47–1.05)
M 5: Adjusted for age, PA, and smoking	**0.46 (0.32–0.66)**	**0.60 (0.38–0.94)**
Walking speed, per 0.1 m/s		
M 1: Adjusted for age	**0.83 (0.77–0.89)**	**0.90 (0.84–0.96)**
M 2: Adjusted for age, height, and weight	**0.82 (0.76–0.89)**	**0.89 (0.83–0.96)**
M 3: Adjusted for age and education	**0.83 (0.77–0.89)**	**0.90 (0.84–0.97)**
M 4: Adjusted for age and coding task	**0.83 (0.77–0.90)**	0.94 (0.87–1.02)
M 5: Adjusted for age, PA, and smoking	**0.80 (0.73–0.87)**	**0.91 (0.84–0.98)**

*HR* hazard ratio, *CI* confidence interval, *M* model, *PA* physical activity, in the 1989–1990 cohort, *n* = 164 and in the 2017–2018 cohort, *n* = 392

Statistically significant values (*p* <.05) are shown in bold

**Table 5:** Associations of muscle strength and walking speed with 5-year mortality in two birth cohorts among women

	2017–2018 cohort	1989–1990 cohort
	HR (95% CI)	HR (95% CI)
Grip strength, per 100 N		
M 1: Adjusted for age	1.13 (0.47–2.70)	**0.57 (0.37–0.86)**
M 2: Adjusted for age, height, and weight	0.97 (0.39–2.40)	**0.57 (0.37–0.89)**
M 3: Adjusted for age and education	1.16 (0.48–2.76)	**0.57 (0.37–0.89)**
M 4: Adjusted for age and coding task	1.06 (0.43–2.59)	**0.69 (0.44–1.08)**
M 5: Adjusted for age, PA, and smoking	1.28 (0.53–3.16)	**0.58 (0.37–0.90)**
Knee extension strength, per 100 N		
M 1: Adjusted for age	**0.56 (0.32–0.98)**	**0.49 (0.34–0.70)**
M 2: Adjusted for age, height, and weight	**0.52 (0.30–0.91)**	**0.48 (0.32–0.70)**
M 3: Adjusted for age and education	0.57 (0.32–1.00)	**0.49 (0.34–0.71)**
M 4: Adjusted for age and coding task	0.61 (0.35–1.09)	**0.57 (0.37–0.84)**
M 5: Adjusted for age, PA, and smoking	0.60 (0.33–1.08)	**0.52 (0.36–0.76)**
Walking speed, per 0.1 m/s		
M 1: Adjusted for age	0.88 (0.77–1.00)	**0.85 (0.80–0.92)**
M 2: Adjusted for age, height, and weight	0.88 (0.76–1.01)	**0.84 (0.78–0.91)**
M 3: Adjusted for age and education	0.87 (0.77–1.00)	**0.87 (0.81–0.93)**
M 4: Adjusted for age and coding task	0.87 (0.77–1.01)	**0.87 (0.81–0.94)**
M 5: Adjusted for age, PA, and smoking	0.90 (0.78–1.04)	**0.86 (0.79–0.94)**

*HR* hazard ratio, *CI* confidence interval, *M* model, *PA* physical activity, in the 1989–1990 cohort, *n* = 392 and in the 2017–2018 cohort, *n* = 518

Statistically significant values (*p* <.05) are shown in bold

Better knee extension strength was associated with mortality reduction in both cohorts of women but in the later born women, the association vanished when adjusted for education, coding task, physical activity, and smoking. Better grip strength and faster walking speed were not statistically significantly associated with mortality reduction in the later born women like they were in the earlier born female cohort. However, the hazard ratios were similar to those of women born earlier except for the grip strength, which was above one. In women born earlier, adjusting for the coding task attenuated the association between muscle strength and mortality as in earlier born men, but not between walking speed and mortality. The birth cohort-by-muscle strength or birth cohort-by-walking speed interactions were not statistically significant in either sex (Supplemental Table 3).

## Discussion

The main results were that the associations of grip and knee extension strength with walking speed were significant and rather similar in the later born cohort of 75- and 80-year-old people when compared to those of the same age born 28 years earlier. The associations of muscle strength and walking speed with mortality were slightly stronger in the more recent compared to earlier cohort of men. However, in more recently born women, the associations were not significant, which may be explained by their low mortality rate and lack of power in the models. The higher values in grip strength, knee extension strength and walking speed in the later born cohorts and lower mortality may denote a larger proportion of older people today have their physical functioning above a critical reserve capacity threshold for sustaining health than before.

A novel finding of this study was that the muscle strength and walking speed distributions of the two cohorts were partly located at different parts of the regression line, but the slopes were primarily parallel. A higher proportion of men born later than those born earlier were above the reserve capacity threshold, especially for lower limb muscle strength. Thus, the curvilinear model including the quadratic term fitted better for explaining the association in more recently born men whereas it did not have additional value over the linear model in the earlier born men, most of whom had lower strength. Similar differences between the cohorts were not observed for the relationship between grip strength and walking speed, which may be explained by the fact that although grip strength represents overall skeletal muscle mass and strength, lower extremity strength is more relevant for walking ability and more prone to lifestyle influences, such as physical activity, than upper extremity strength [18, 19]. Thus, muscle strength reserve for walking may not be as clearly reflected in upper compared to lower extremity muscles. Our results are in line with a previous study reporting men with a flattening lower extremity strength-maximal walking speed relationship at higher strength levels and women without a plateau [5].

Women generally have less muscle strength compared to men, which explains the clearer linear relationship at older age. However, the observed group-level difference in the associations suggest that the muscle strength needed for maintaining walking ability may have decreased over time. For example, the women of the earlier born cohort reached the 1.22 m/s walking speed, which has been used as a cut-off to cross the street in signaled intersections [2, 20] with about 150 Newtons in grip and knee extension strength. With the same level of muscle strength, the women of the later born cohort reached a walking speed of 1.5 m/s. This difference is most likely significant as already a 0.1 m/s difference in walking speed has been shown to translate into better mobility [21] implying that older adults nowadays may achieve adequate walking speed with less muscle strength than before. These differences between the birth cohorts were also partly explained by their differences in education, physical activity, smoking and cognitive functioning. It is also possible that the presence of other health resources, which we were not able to control for, such as better cardiovascular fitness or motor control affecting e.g., balance and stride length or lower prevalence of painful conditions in legs, help later born older adults to maintain mobility also at lower muscle strength levels.

Interestingly, among later born men, the associations of muscle strength and walking speed with mortality were not explained by the covariates, especially coding task, in the same way as in the earlier born cohort. It is possible that the accelerated terminal decline in cognition and other health domains that tend to occur during the last years of life [22] may not have been prevailing yet among the later born men, who were further away from death than their earlier born counterparts. This is supported by the fact that compared to earlier born cohort the later born cohort had better cognitive functioning but also longer education, and larger body size, which reflect a variety of more favorable life-course exposures that may help to resist the acceleration of health deficit accumulation and terminal decline.

In later born women, who had the lowest mortality rate, the associations of muscle strength and walking speed with mortality were mostly not significant, which is most likely explained by low statistical power. However, it was notable that grip strength did not differ at all between the deceased and survivors, which may indicate that its role as an indicator of physiological reserve and a predictor of mortality may have altered among later born women. This could stem from, for example, changes in work history and daily life activities, which have not strained the muscles of the upper extremities in the same way as before. In further analyses, we found that the correlation between grip strength and knee extension strength was somewhat weaker in later born compared to earlier born women (r = 0.506 vs. 0.703), which supports the hypothesis that in women, grip strength as a proxy of overall body muscle strength may have weakened. In men, the correlation between the strength measurements remained rather similar (r = 0.502 vs. 0.588 in later and earlier born men, respectively).

Greater functional reserve capacity and possible postponement of mobility and health decline in the later born older adults may stem from many positive societal changes during the past century, which have ensured more propitious life-course exposures for the later born cohorts. The earlier cohorts of the current study were born in 1910–1915 and when Finland was an agricultural society, where children started working from early age. The earlier born cohort also experienced the Civil war in 1918 and participated in the Winter War (1939–40) and the Continuation War (1941–1944) as young adults. The later born cohorts of this study were also born during the war years, but they had the opportunity to benefit from many reforms and rapid development of the post-war Finnish society later in their early life, which undoubtedly had a positive effect on their health and functioning. We also observed signs of these societal changes between the birth cohorts in our sample. For example, men and women born later had longer education, reported more leisure-time physical activity, and had better cognitive functioning, which also partly explained the observed birth cohort differences in the associations between muscle strength, walking speed and mortality.

Our study has many strengths that give us confidence that the observed results are real and do not result from methodological limitations. The two birth cohorts of this study are based on representative population-based samples with identical recruitment procedures and assessed using the same standardized physical performance measurements. This allowed us to compare the associations between muscle strength and walking speed, and the associations of both of these with mortality between the cohorts reliably. The current results are also unique in that they are based not only on grip strength but also on lower extremity muscle strength and walking speed, which are much less commonly measured in epidemiological studies using standardized methods across cohorts but which may better describe functional capacity needed for daily functioning [23]. In addition, we used mortality as an outcome, which is probably the most reliably measurable indicator of health deterioration [24], and the comparison of mortality rates over time from the Finnish Population Register is very accurate, as it is continuously maintained and updated by the Population Register authorities.

It is also important to address some limitations. The participation rate was lower in the later than in the earlier cohort, which could mean that the participants in the more recent study were more selected. However, the earlier born cohort was representing the surviving elite of their generation while in the later born cohort, more individuals with diseases and disabilities may have survived into the old age. This may attenuate the observed cohort differences in the physical performance levels and the studied associations between the cohorts. In addition, we did not observe substantial differences in the reasons for not participating in the study between the cohorts [10], which supports the comparability of the cohorts. In addition, the cohort and sex groups may have been too small, and subsequently, our models underpowered to detect statistically significant differences in the associations between muscle strength, walking speed, and mortality, although the results of the stratified analyses gave indication of these differences. It is also worth noting that full-time education may not capture all aspects of socioeconomic status but was selected as a covariate as it is a more comparable variable between different birth cohorts than, for example, income level. Finally, the results may be specifically generalized to Finnish population, although it is very likely that similar results can be found in other countries that have experienced similar social changes during the last decade.

In conclusion, muscle strength remains a significant predictor of walking speed among the newer cohorts of older adults who, however, seem to have more functional reserve capacity to maintain walking ability for longer. The mortality risk associated with lower muscle strength and walking speed may have slightly decreased among women at ages 75–80.

### Supplementary Information

Below is the link to the electronic supplementary material.

Supplementary file1 (DOCX 31 KB)

## Data Availability

Pseudonymized data are available to external collaborators upon agreement on the terms of data use and publication of results. To request the data, please contact Professor Taina Rantanen (taina.rantanen@jyu.fi).
