# Supplementary material for: Comparing the associations between muscle strength, walking speed, and mortality in community-dwelling older adults of two birth cohorts born 28 years apart
Source: GeroScience. 2023 Sep 1;46(2):1575–88. doi: 10.1007/s11357-023-00925-z (PMC10828148; doi:10.1007/s11357-023-00925-z)
Supplement: Supplementary file 1 — Supplementary file1 (DOCX 31 KB) [file 11357_2023_925_MOESM1_ESM.docx]

Supplemental information

**Comparing the associations between muscle strength, walking speed, and mortality in community-dwelling older adults of two birth cohorts born 28 years apart**

Kaisa Koivunen, Erja Portegijs., Laura Karavirta, & Taina Rantanen

Corresponding author:

Kaisa Koivunen,

Faculty of Sport and Health Sciences and Gerontology Research Center, the University of Jyväskylä, Finland

E-mail: kaisa.m.koivunen@jyu.fi

Supplemental Table 1. Linear and curvilinear associations between grip strength and walking speed in a sample combining the birth cohorts.

|  |  | Unadjusted | |  | Adjusted | |
| --- | --- | --- | --- | --- | --- | --- |
| **Men** |  | **B (SE)** | **p** |  | **B (SE)** | **p** |
| M1 | Cohort | .152 (.043) | **<.001** |  | -.037 (.507) | .442 |
|  | Grip strength (linear) | .218 (.024) | **<.001** |  | .159 (.025) | **<.001** |
|  | **R^2^** | *.193* |  |  | *.391* |  |
| M2 | Cohort | .137 (.043) | **.001** |  | -.046 (.048) | .338 |
|  | Grip strength (linear) | .596 (.137) | **<.001** |  | .356 (.126) | **.005** |
|  | Grip strength (quadratic) | -.050 (.018) | **.005** |  | -.026 (.017) | .113 |
|  | **R^2^** | *.205* |  |  | *.393* |  |
| M3 | Cohort | .297 (.185) | .110 |  | -.045 (.172) | .791 |
|  | Grip strength (linear) | .280 (.082) | **.001** |  | .155 (.076) | **.043** |
|  | Grip (linear) * cohort | -.040 (.049) | .421 |  | .002 (.044) | .959 |
|  | **R^2^** | *.192* |  |  | *.389* |  |
| **Women** | |  |  |  |  |  |
| M1 | Cohort | .245 (.024) | **<.001** |  | .137 (.027) | **<.001** |
|  | Grip strength (linear) | .249 (.021) | **<.001** |  | 204 (.020) | **<.001** |
|  | **R^2^** | *.294* |  |  | *.495* |  |
| M2 | Cohort | .242 (.024) | **<.001** |  | .136 (.028) | **<.001** |
|  | Grip strength (linear) | .324 (.108) | **.003** |  | .257 (.096) | **.007** |
|  | Grip strength (quadratic) | -.017 (.024) | .481 |  | -.012 (.021) | .570 |
|  | **R^2^** | *.294* |  |  | *.494* |  |
| M3 | Cohort | .523 (.143) | **<.001** |  | .094 (.086) | .279 |
|  | Grip strength (linear) | .299 (.065) | **<.001** |  | .175 (.059) | .003 |
|  | Grip (linear) * cohort | -.034 (.043) | .419 |  | .020 (.037) | .597 |
|  | **R^2^** | *.294* |  |  | *.494* |  |

Note. B= unstandardized regression coefficient (per -100 Newtons in grip strength), SE = standard error, M1 (model 1): cohort variable + linear term of grip strength, M2 (model 2) = M1 + quadratic term of grip strength, M3 (model 3) = M1 + interaction between cohort and grip strength, covariates in the adjusted models: age group, height, weight, education, physical activity, smoking, and coding task; adjusted R^2^ = adjusted, the variance explained by the models.

Supplemental Table 2. Linear and curvilinear associations between knee extension (KE) strength and walking speed in a sample combining the birth cohorts.

|  |  | Unadjusted | |  | Adjusted | |
| --- | --- | --- | --- | --- | --- | --- |
| **Men** |  | **B (SE)** | **p** |  | **B (SE)** | **p** |
| M1 | Cohort | .080 (.044) | .070 |  | -.093 (.048) | .056 |
|  | KE strength (linear) | .193 (.020) | **<.001** |  | .141 (.018) | **<.001** |
|  | **R^2^** | *.216* |  |  | *.406* |  |
| M2 | Cohort | .076 (.043) | .078 |  | -.089 (.048) | .064 |
|  | KE strength (linear) | .566 (.101) | **<.001** |  | .419 (.096) | **<.001** |
|  | KE strength (quadratic) | -.045 (.012) | **<.001** |  | -.033 (.011) | **.003** |
|  | **R^2^** | *.239* |  |  | *.416* |  |
| M3 | Cohort | .626 (.168) | **<.001** |  | .269 (.159) | .092 |
|  | KE strength (linear) | .748 (.132) | **<.001** |  | .307 (.072) | **<.001** |
|  | KE (linear) * cohort | -.243 (.074) | **.001** |  | -.095 (.040) | **.018** |
|  | **R^2^** | *.229* |  |  | *.412* |  |
| **Women** | |  |  |  |  |  |
| M1 | Cohort | .173 (.026) | **<.001** |  | .078 (.028) | **.004** |
|  | KE strength (linear) | .187 (.015) | **<.001** |  | .161 (.014) | **<.001** |
|  | **R^2^** | *.309* |  |  | *.517* |  |
| M2 | Cohort | .172 (.026) | **<.001** |  | .077 (.028) | **.005** |
|  | KE strength (linear) | .264 (.062) | **<.001** |  | .205 (.054) | **<.001** |
|  | KE strength (quadratic) | -.014 (.011) | .207 |  | -.008 (.010) | .399 |
|  | **R^2^** | *.309* |  |  | *.516* |  |
| M3 | Cohort | .262 (.082) | **.001** |  | .136 (.072) | .061 |
|  | KE strength (linear) | .376 (.083) | **<.001** |  | .199 (.046) | **<.001** |
|  | KE (linear) * cohort | -.050 (.049) | .303 |  | -.022 (.027) | .394 |
|  | **R^2^** | *.305* |  |  | *.516* |  |

Note. KE = knee extension, B= unstandardized regression coefficient (per -100 Newtons in KE strength), SE = standard error, M1 (model 1): cohort variable + linear term of KE strength, M2 (model 2) = M1 + quadratic term of KE strength, M3 (model 3) = M1 + interaction between cohort and KE strength, covariates in the adjusted models: age group, height, weight, education, physical activity, smoking, and coding task; adjusted R^2^ = adjusted, the variance explained by the models.

Supplemental Table 3. Main and interaction effects of muscle strength, walking speed and birth cohort on 5-year mortality in a sample combining the birth cohorts.

| **Men** |  | HR (95% CI) |
| --- | --- | --- |
|  | **Grip strength** |  |
| M1 | Cohort | 0.82 (0.49-1.37) |
|  | Grip strength, per 100 N | **0.49 (0.36-0.67)** |
| M2 | Cohort | 1.12 (0.14-8.57) |
|  | Grip strength, per 100 N | 0.56 (0.23-1.36) |
|  | Cohort * grip strength | 0.91 (0.50-1.66) |
|  | **Knee extension strength** |  |
| M1 | Cohort | 1.00 (0.59-1.68) |
|  | Knee extension strength, per 100 N | **0.49 (0.38-0.64)** |
| M2 | Cohort | 1.86 (0.30-11.41) |
|  | Knee extension strength, per 100 N | 0.66 (0.27-1.60) |
|  | Cohort * knee extension strength | 0.83 (0.49-1.41) |
|  | **Walking speed** |  |
| M1 | Cohort | 0.95 (0.56-1.58) |
|  | Walking speed, per 0.1 m/s | **0.86 (0.82-0.91)** |
| M2 | Cohort | 3.44 (0.73-16.20) |
|  | Walking speed, per 0.1 m/s | 0.98 (0.84-1.14) |
|  | Cohort * walking speed | 0.92 (0.83-1.01) |
| **Women** |  |  |
|  | **Grip strength** |  |
| M1 | Cohort | **0.28 (0.17-0.46)** |
|  | Grip strength, per 100 N | **0.62 (0.42-0.91)** |
| M2 | Cohort | **0.05 (0.01-0.46)** |
|  | Grip strength, per 100 N | **0.25 (0.08-0.21)** |
|  | Cohort * grip strength | 2.15 (0.84-5.49) |
|  | **Knee extension strength** |  |
| M1 | Cohort | **0.38 (0.22-0.65)** |
|  | Knee extension strength, per 100 N | **0.50 (0.37-0.68)** |
| M2 | Cohort | **0.24 (0.05-1.21)** |
|  | Knee extension strength, per 100 N | **0.38 (0.16-0.94)** |
|  | Cohort * knee extension strength | 1.23 (0.64-2.37) |
|  | **Walking speed** |  |
| M1 | Cohort | **0.41 (0.24-0.70)** |
|  | Walking speed, per 0.1 m/s | **0.86 (0.81-0.91)** |
| M2 | Cohort | 0.20 (0.02-1.83) |
|  | Walking speed, per 0.1 m/s | **1.03 (0.65-1.62)** |
|  | Cohort * walking speed | 1.05 (0.91-1.21) |

Note. N = Newtons, HR = hazard ratio, CI = confidence interval, M1 = model 1 includes the main effects of muscle strength / walking speed and cohort; M2 = M1 + interaction between cohort and muscle strength/walking speed, all models adjusted for age group.
